# Incidence and Prevalence of Poststroke Shoulder Pain Among Different Regions of the World: A Systematic Review and Meta-Analysis

**DOI:** 10.3389/fneur.2021.724281

**Published:** 2021-11-04

**Authors:** Qian Zhang, Danna Chen, Yuxian Shen, Minjie Bian, Pu Wang, Jun Li

**Affiliations:** ^1^Department of Rehabilitation Medicine, The Seventh Affiliated Hospital, Sun Yat-Sen University, Shenzhen, China; ^2^Department of Urology, The Seventh Affiliated Hospital, Sun Yat-Sen University, Shenzhen, China

**Keywords:** poststroke shoulder pain, incidence, prevalence, meta-analysis, epidemiology

## Abstract

**Objectives:** Poststroke shoulder pain (PSSP) is a common complication after stroke. This review aimed to provide updated information on the epidemiological characteristics of PSSP, reveal their trends over time and region.

**Study Design and Setting:** We searched the PubMed, Embase, Cochrane Library and Web of Science databases from inception until Dec 31, 2020. Data were extracted from the eligible studies, and their quality was assessed. The pooled incidence and prevalence estimates of PSSP and their 95% confidence intervals (CIs) were calculated. We analyzed the incidence and prevalence of PSSP by different geographical regions and countries and separately calculated the annual incidence and prevalence (and 95% CIs) of PSSP.

**Results:** A total of 21 studies were eligible for the meta-analysis. Eleven cohort studies were included to analyze the incidence of PSSP, and the estimated pooled incidence in 3,496 stroke patients was 0.29 (95% CI 0.21–0.36). Ten cross-sectional studies were included to analyze the prevalence of PSSP, and the pooled prevalence in 3,701 stroke patients was 0.33 (95% CI 0.22–0.43). In addition, we found that there were significant differences in the incidence and prevalence of PSSP between different geographical regions and different countries. Additionally, the incidence of PSSP fluctuated around 30%, and the prevalence had a downward trend over time.

**Conclusions:** Current evidence suggests that the incidence and prevalence of PSSP are high and may be influenced by geographical region and time.

## Introduction

Poststroke shoulder pain (PSSP) is a common complication that usually appears within 2 weeks−2 months after the occurrence of stroke (1, 2). It has a negative effect on the recovery of motor and sensory function and seriously affects the quality of life of patients (3). Additionally, PSSP can lead to psychological changes and sleep disorders, including depression, anxiety, fear avoidance, hypervigilance, pain catastrophizing, difficulty falling asleep, early awakening (4, 5). Consequently, it is closely related to the prolonged hospital stay (6, 7) as well as the increased costs of care, and is a significant source of burden to patients' families and communities (8).

To date, the risk factors and pathological mechanisms associated with PSSP have yet to be fully elucidated. There are not only motor control impairments (changes in muscle tone) and peripheral and central nervous activity changes but also soft tissue injuries (9, 10). These may occur separately or simultaneously. In addition, each pathology may initiate the development of another. It remains unclear how these pathologies may interact or correlate with each other. Based on the multifactorial etiology and risk factors of PSSP, many interventions have been developed in the past few decades. Pomeroy et al. conducted semi-structured and medical interviews in the United Kingdom and found that 175 different types of interventions for PSSP have been used (11). Snels et al. conducted postal questionnaires with structured and open-ended questions in the Netherlands and found 54 different combinations of therapeutic schedules (12). Main interventions include electrical stimulation, acupuncture, pharmaceutical therapies, kinesiotherapy and best nursing practices. The most common first choice of treatment was physiotherapy (32%), which included neuromuscular electrical stimulation (NMES) and transcutaneous nerve stimulation (TENS) (13).

With the medicine information and techniques continuously updating and changing results of etiology, associated risk factors and interventions, the incidence and prevalence of PSSP will need to be reappraised in the context of changing epidemiological patterns worldwide. However, there was no comprehensive systematic review of epidemiology of PSSP up to now. Therefore, we performed a systematic review of cohort studies and cross-sectional studies reporting the incidence and prevalence of PSSP based on different geographical regions and annual trends. We aimed to provide updated information on the epidemiological characteristics of PSSP and reveal their trends over time and region. In the future, our team will summarize the epidemiological characteristics of all poststroke complications.

## Methods

This study was reported according to the Preferred Reporting Items for Systematic Reviews and Meta-Analyses guidelines (Supplementary Table 1) and the Meta-analysis of Observational Studies in Epidemiology Guidelines Checklist (Supplementary Table 2).

### Patient and Public Involvement

Patients and/or the public were not involved in the design, or conduct, or reporting, or dissemination plans of this research.

### Search Strategy

We searched the PubMed, Embase, Cochrane Library and Web of Science databases for observational studies from inception until Dec 31, 2020. No language restrictions were applied. The search strategy used both Medical Subject Headings (MeSH) and keyword terms, including prevalence, incidence, and epidemiology in conjunction with hemiplegia or post-stroke shoulder pain. The full search strategy is shown in the Supplementary Table 3. The references listed in the included articles were also manually searched.

### Inclusion and Exclusion Criteria

We included epidemiologic studies that quantitatively examined the incidence and/or prevalence of PSSP using cross-sectional or longitudinal observational study designs. Studies were excluded if they had the interventions to the PSSP patients, if they used incomplete or duplicative data or if they were review articles, abstracts or editorials, or articles in press.

### Study Selection Process

Two authors (Qian Zhang and Yuxian Shen) independently screened and extracted data from the included literature, input the data into in a standardized Microsoft Excel spreadsheet and crosschecked the other's work. Any disagreement was resolved by consensus with a third author (Jun Li). If necessary, we contacted the authors of eligible studies by email or phone to obtain information that was not described in their study but was important for our analyses. All studies were downloaded into Endnote X9 software and de-duplicated, selected first on the basis of title and abstract and then for the full text.

We categorized the incidence and/or prevalence data by geographical region using the United Nations classification of economic regions (14), which is based on geographical proximity and economic similarities. The regions are North America, Europe (northern, southern, western, eastern), Africa, Asia (eastern, southern, southeastern, western), South America, and Oceania. We extracted the following information: (1) basic information, including geographical region, year, region, recruitment site, sponsorship, design, sample, proportion of females, and mean age; (2) baseline characteristics of stroke and PSSP patients, including type of stroke, diagnostic criteria, location, disease time, PSSP assessment, PSSP sample, hemiplegia, mean age, and follow-up time or investigation period; and (3) outcome indicators and outcome measurement data that we needed.

### Study Outcomes

The primary outcomes were the pooled incidence and prevalence rate of PSSP worldwide. In addition, we analyzed the incidence or prevalence of PSSP by different geographical regions and countries. Then, we separately calculated the annual incidence and prevalence rates (with the 95% CIs) of PSSP.

We defined the following information: (1) incidence rate refers to the frequency of new cases of a disease in a particular population at a particular time; (2) prevalence rate is defined as the proportion of new and existing cases of a disease in a particular population at a particular time; (3) annual incidence rate is the incidence rate in a year; (4) annual prevalence rate is the prevalence rate in a year.

### Assessment of Study Quality

The methodological quality of case-control studies and cohort studies was evaluated using the Newcastle-Ottawa Scale (NOS) (15). The NOS assigns a maximum of nine points to three quality parameters, i.e., selection, comparability, and outcome or exposure, which are evaluated across eight items. The quality of the cross-sectional studies was assessed by the Agency for Healthcare Research and Quality (AHRQ) tool (16). The AHRQ tool consists of 11 items, including the source of information, inclusion and exclusion criteria, time period, consecutive or population-based study subjects, evaluators of subjective components, quality control, etc., with a total score of 11. Two authors independently evaluated the quality of the selected studies. A third investigator resolved any disagreements.

### Statistical Analysis

Data analysis was performed using STATA 15 software. We computed the pooled incidence and prevalence rate with 95% CIs. The Q test and I^2^ statistic were used to assess heterogeneity. If heterogeneity was present (*P* < 0.1, I^2^ ≥ 50%), the random effects model was selected, and subgroup analysis was used to identify the source of heterogeneity. Additionally, sensitivity analyses were used to assess the robustness and stability of the results. We constructed funnel plots and observed whether they are symmetrical to qualitatively assess publication bias. Furthermore, Begg's test and Egger's test were used to quantitatively examine publication bias. A significance level of 0.05 was used.

## Results

**Figure 1:**
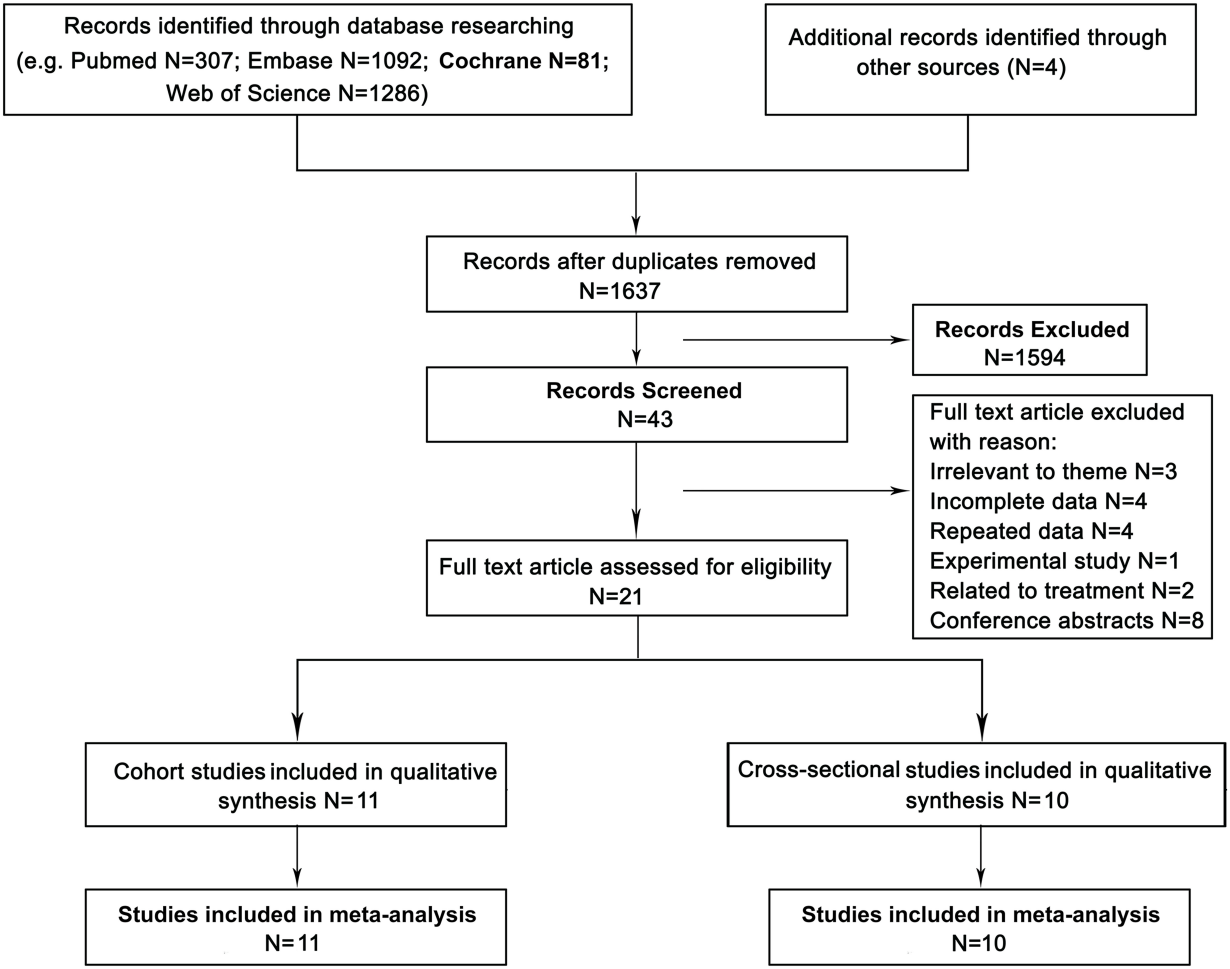
Flow-chart of studies' selection process.

The search identified a total of 2,766 articles from the databases and 4 studies retrieved from our manual search. After 1,125 duplicates were removed, 1,637 studies remained for title and abstract screening. Subsequently, 1,594 articles were eliminated, and the full texts of the remaining 43 studies were examined. Twenty studies were then excluded for reasons such as having irrelevant to theme (*N* = 3), having incomplete data (*N* = 4), having repeated data (*N* = 4), having related to treatment (*N* = 2), and being experimental study (*N* = 1) or conference abstracts (*N* = 8). Ultimately, 21 studies (17–37), including 11 cohort studies (17–27) and 10 cross-sectional studies (28–37), were included in the meta-analysis (Figure 1).

### Quality Evaluation of Included Studies

The quality of the included studies was evaluated in the Tables 1, 2. The overall quality of the eligible articles was good. The NOS score ranged from 6 to 9 for the cohort studies (Table 1), while the AHRQ score ranged from 10 to 11 for the cross-sectional studies (Table 2).

**Table 1:** Bias of included cohort studies.

**References**	**Selection**				**Comparability**		**Outcome**		**Total scare**
	**Representativeness of the exposed cohort**	**Selection of the non exposed cohort**	**Ascertainment of exposure**	**Demonstration that outcome of interest was not present at start of study**	**Comparability of cohort on the basis of the design or analysis**	**Assessment of outcome**	**Was follow-up long enough for outcomes to occur**	**Adequacy of follow up of cohorts**	
Davenport et al. (18)	1	1	0	1	2	0	1	1	7
Wanklyn et al. (19)	1	1	1	1	1	1	1	1	8
Langhorne et al. (38)	1	1	0	1	1	0	1	1	6
Gamble et al. (21)	1	1	1	1	2	1	1	1	9
Ratnasabapathy et al. (26)	1	1	1	1	2	1	1	1	9
McLean (17)	1	1	0	1	2	0	1	1	7
Lindgren et al. (24)	1	1	1	1	2	1	1	1	9
Sackley et al. (22)	1	1	1	1	2	1	1	1	9
Roosink et al. (25)	1	1	1	1	2	1	1	1	9
Adey-Wakeling et al. (27)	1	1	1	0	2	1	1	1	8
Nadler et al. (23)	1	1	1	0	2	1	0	1	7

**Table 2:** Bias of included cross-sectional studies.

**References**	**Define the source of information (survey, record review)**	**List inclusion and exclusion criteria for exposed and unexposed subjects (cases and controls) or refer to previous publications**	**Indicate time period used for identifying patients**	**Indicate whether or not subjects were consecutive if not population-based**	**Indicate if evaluators of subjective components of study were masked to other aspects of the status of the participants**	**Describe any assessments undertaken for quality assurance purposes (e.g., test/retest of primary outcome measurements)**	**Explain any patient exclusions from analysis**	**Describe how confounding was assessed and/or controlled**	**If applicable, explain how missing data were handled in the analysis**	**Summarize patient response rates and completeness of data collection**	**Clarify what follow-up, if any, was expected and the percentage of patients for which incomplete data or follow-up was obtained**	**Total scare**
Aras et al. (24)	1	1	1	1	1	1	1	1	1	1	1	11
Dromerick et al. (29)	1	1	1	1	1	1	1	0	1	1	1	10
Barlak et al. (31)	1	1	1	1	1	1	1	1	1	1	1	11
Lundström et al. (33)	1	1	1	1	1	1	1	1	1	1	1	11
Hamzat and Osundiya (28)	1	1	1	0	1	1	1	1	1	1	1	10
Klit et al. (34)	1	1	1	1	1	1	1	1	1	1	1	11
Kwon et al. (32)	1	1	1	0	1	1	1	1	1	1	1	10
Paolucci et a. (35)	1	1	1	1	1	1	1	1	1	1	1	11
Janus-Laszuk et al. (36)	1	1	1	1	1	0	1	1	1	1	1	10
Menoux et al. (37)	1	1	1	0	1	1	1	1	1	1	1	10

### Participant Characteristics

The characteristics of cohort studies were summarized in Table 3. According to the available data, the stroke patients were mostly elderly people with cerebral infarction in the acute phase. The sample sizes ranged from 31 (25) to 1,474 (26). Three studies (24, 26, 27) (27.3%) were population-based. PSSP patients' pain assessments were mostly conducted using the Visual Analog Scale (VAS) (21, 24, 27), the Numeric Rating Scale (NRS) (23, 25), and questionnaires (19, 21). Most of the follow-up times were longer than 1 month.

**Table 3:** Characteristics of cohort studies.

**Geographical region**	**Region**	**References**	**Sponsor-ship**	**Design**	**Total sample**	**Female**	**Mean age**	**Stroke**					**PSSP**					**Follow-up time**
								**Cerebral infarction/hemorrhage**	**Stroke diagnostic criteria**	**Location (left/right/both)**	**Stroke subtypes**	**Disease time**	**Assessment**	**Sample (female)**	**Mean age**	**Hemiplegia (left/right)**	**Stroke subtypes**	
America	Canada	McLean et al. (17)	*	prospective cohort study	133	64	68.6 ± 13.1	109/24	*	64/60/8	*	after stroke	Self-reported	32(*)	*	*	*	12-month
Europe	United Kingdom of Great Britain and Northern Ireland(UK)	Davenport et al. (18)	Yes	prospective cohort study	607	328	73	550/57	the WHO criteria	*	*	after stroke	*	27	*	*	*	30-day
		Wanklyn et al. (19)	Yes	cohort study	112	*	*	*	the WHO criteria	*	*	after stroke	interview-administered questionnaire	59	*	*	*	8-week
					108	51	71			57/46/5				36				6-month
		Langhorne et al. (19)	Yes	cohort study	220	*	76	*	the WHO criteria	*	*	<7 days	*	33	*	*	*	6-month
					181	*								20				18-month
					155	*								19				30-month
		Gamble et al. (2)	Yes	cohort study	123	66	70.6	119/4	the WHO criteria	*	*	<5 days	questionnaire of pain history/VAS	52 (34)	68	*	*	6-month
		Sackley et al. (22)	Yes	cohort study	122	53	76 ± 11	*	*	*	*	88 ± 62 days	clinical assessment by staff/caregiver/physiotherapist	44 (*)	*	*	*	3-month
					89	*	*							37 (*)				6-month
					73	*	*							34 (*)				12-month
		Nadler et al. (23)	Yes	observational, prospective cohort study	121	53	69 ±13	103/18	CT/MRI/clinical examination	67/54	*	72 h	NRS	53	*	21/32	*	8–10 weeks
	Sweden	Lindgren et al. (24)	Yes	prospective population-based cohort study	327	132	73.1	314/13/*	the WHO criteria/CT	*	LACS(105)/PACS(117)/POCS(54)/TACS(38)/Subarachnoid hemorrhage (13)	after stroke	VAS	71 (26)	72.3	*	LACS(101)/PACS(108)/POCS(52)/TACS(31)/Subarachnoid hemorrhage (13)	4-month
					305	123	72.5	292/13/*						74 (27)	72.2	*		16-month
	Netherlands	Roosink et al. (25)	Yes	prospective cohort study	31	17	52~82	31/*/*	a clinical diagnosis	11/20/*	*	after stroke	a pain diagram/numeric rating scale (NRS)	11 (*)	*	*	*	3-month
														9 (*)	72 ± 10	1/8		6-month
Oceania	New Zealand	Ratnasabapathy et al. (26)	Yes	population-based cohort study	1,474	773	*	*	the WHO criteria	110/121	*	after stroke	Self-reported	256(138)	*	79/102	*	1-week
					1,336	692	*			119/120				261(132)	*	96/106		1-month
					1201	580	*			144/124				284(132)	*	104/109		6-month
	Australia	Adey-Wakeling et al. (27)	Yes	prospective population-based cohort study	226	71	pain:73 ± 15; no pain: 72 ± 14;	200/20/*	*	122/104	LACS(54)/PACS(89)/ POCS(37)/TACS(40)/Unknown(6)	8.7 days	Self-reported/VAS	65	72 ± 14	21/44	LACS(19)/PACS(22)/POCS(9)/TACS(14)/Unknown (1)	12-month

VAS, Visual Analog Scale; BNS, Numerical Box-21 Scale; NRS, Numeric Rating Scale; NPRS, Numerical Point Rating Scale; SPADI, Shoulder Pain and Disability Index; PPTs, Pressure Pain Thresholds; LACS, Lacunar Syndromes; PACS, Partial Anterior Circulation Syndrome; POCS, Posterior Circulation Syndrome; TACS, Total Anterior Circulation Syndrome; *, unclear.

While, the characteristics of cross-sectional studies were summarized in Table 4. The mean age of the patients in these studies was younger than that of patients from the cohort studies, and most of these patients were in the recovery or sequela phases. The sample sizes ranged from 46 (29) to 1,075 (36). Only one study (34) (10.0%) was population-based. The pain of the PSSP patients was assessed using scales or clinical examinations, but one study (36) used self-reported only. The survey periods ranged from 1 year (29, 32–34) to 5 years (37).

**Table 4:** Characteristics of cross-sectional studies.

**Geographical region**	**Region**	**References**	**Sponsor-ship**	**Design**	**Period**	**Total sample**	**Female**	**Mean age**	**Stroke**					**PSSP**				
									**Cerebral infarction/hemorrhage**	**Stroke diagnostic criteria**	**Location (left/right/both)**	**Stroke subtypes**	**Disease time**	**Assessment**	**Sample (female)**	**PSSP mean age**	**Hemiplegia (left/right)**	**Stroke subtypes**
Africa	Nigeria	Hamzat and Osundiya (28)	*	prospective cross-sectional survey	*	102	51	52.92 ± 10.24	*	*	*	*	1~15 month	BNS	77(*)	*	*	*
America	United States of America (USA)	Dromerick et al. (29)	Yes	prospective cross-sectional study	2008	46	22	57.30 ± 25.20	*	*	24/22/*	*	18.9 ± 14.1 days	Self-reported/VAS	17(*)	*	*	*8
Asia	Turkey	Aras et al. (24)	*	cross-sectional survey	*	85	26	59.5 ± 11.6	60/25	the WHO criteria	38/47/*	*	<3 month	Medical history	54(16)	61.5 ± 10.3	31/23	*
		Barlak et al. (29)	*	observational cross-sectional study	2005–2007	187	95	36–75	138/49	a clinical diagnosis and CT/MRI	*	*	pain: 124.30 ± 55.10; no pain: 89.93 ± 42.54;	VAS	114(53)	62.35 ± 10.65	6/106	*
	Korea	Kwon et al. (32)	*	observational cross-sectional study	2011	229	96	59.0 ± 12.4	125/28	CT/MRI	107/122	*	2–180 days	the pain diagram	62(34)	61.53 ± 11.07	38/24	*
Europe	Sweden	Lundström et al. (33)	Yes	cross-sectional survey	2003–2004	140	67	71 ± 13	124/16	the WHO criteria	57/77/6	*	>1 year	VAS	9(*)	*	*	*
	Denmark	Klit et al. (34)	Yes	population-based cross-sectional survey	2004–2005	608	268	72.6	*	the WHO criteria	*	*	794.5 days (588–1,099)	questionnaire/NRS	92(*)	*	*	*
	Italy	Paolucci et al. (35)	*	observational cross-sectional study	2010–2012	443	*	acute: 67.16 ± 14.08; subacute: 67.60 ± 14.18; chronic: 66.59 ± 14.73;	acute: 298/22; subacute: 99/11; chronic: 102/14;	clinical examination/neuro-radiological findings	acute: 170/150; subacute: 68/52; chronic: 57/59;	*	acute: 2.10 ± 2.83 days; subacute: 47.77 ± 24.42 days; chronic: 174.89 ± 107.71 days;	clinical history/examination	acute: 3; subacute: 77; chronic: 46;	*	*	*
	Poland	Janus-Laszuk et al. (36)	Yes	observational cross-sectional study	2006–2010	1,075	585	63.96 ± 12.74	876/199	the 2013 WHO criteria/CT/MRI	*	*	100 days	Self-reported	157	*	*	*
	France	Menoux et al. (37)	Yes	retrospective observational cross-sectional study	2000–2015	786	280	58.1 ± 13.2	530/256	*	354/283/47	*	*	Self-reported/examination	32	*	*	*

VAS, Visual Analog Scale; BNS, Numerical Box-21 Scale; ^*^, unclear.

### Meta-Analysis Results

**Figure 2:**
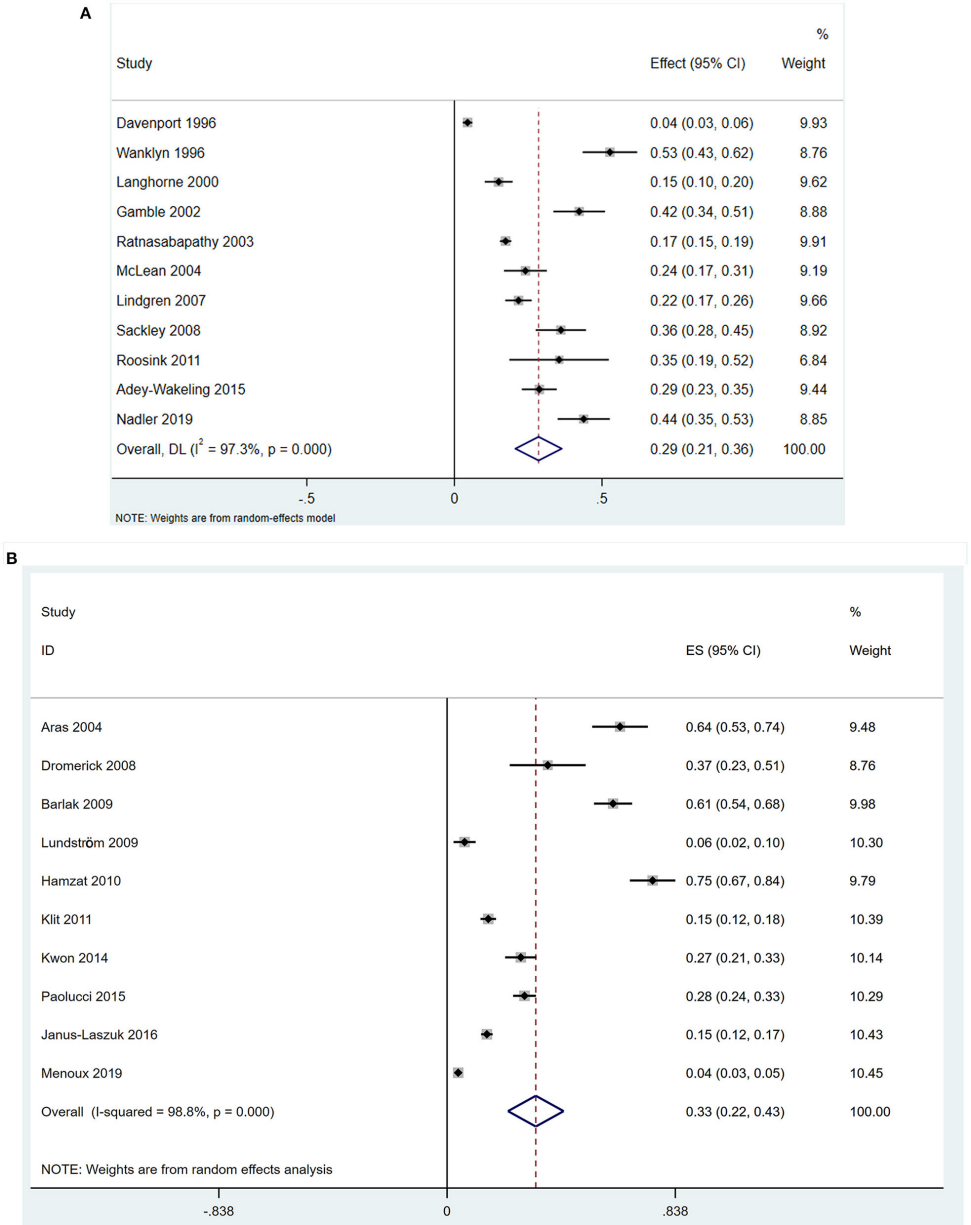
Forest plots. **(A)** Forest plots of incidence of PSSP. CI, confidence interval; DL, DerSimonian-Laird; **(B)** Forest plots of prevalence of PSSP. ES, Effect Size.

The eleven cohort studies assessed the incidence of PSSP. There was significant heterogeneity between the included studies (I^2^ = 97.3 > 50% and the Q test was significant at *P* = 0.000 < 0.1). Therefore, the random effects model was selected for meta-analysis. The pooled incidence rate of PSSP in 3,496 stroke patients was 0.29 (95% CI 0.21–0.36), and the results were statistically significant (*z* = 25.06, *p* = 0.000 < 0.05) (Figure 2A). Sensitivity analysis results indicated that none of the eligible studies were a significant source of heterogeneity, and the accuracy and stability of the results were good (Supplementary Figure 1A). The funnel plots were asymmetric (Supplementary Figure 2A). Furthermore, the results of Begg's test (*P* = 0.0430 < 0.05) and Egger's test (*P* = 0.0020 < 0.05) were significant, indicating that there was publication bias.

The ten cross-sectional studies assessed the prevalence of PSSP. There was significant heterogeneity between the included studies (I^2^ = 98.8 > 50%, and the Q test was significant at *P* = 0.000 < 0.1). Therefore, the random effects model was selected. The pooled prevalence rate of PSSP in 3,701 stroke patients was 0.33 (95% CI 0.22–0.43), and the results were statistically significant (*z* = 6.25, *p* = 0.000 < 0.05) (Figure 2B). Sensitivity analysis results indicated that none of the eligible studies were a significant source of heterogeneity, and the accuracy and stability of the results were good (Supplementary Figure 1B). The funnel plots were asymmetric (Supplementary Figure 2B). Furthermore, the results of Begg's test (*P* = 0.049) and Egger's test (*P* = 0.0020 < 0.05) were significant, indicating that there was publication bias.

### Characteristics of Included Studies in Geographical Region

Table 5 shows the incidence and prevalence of PSSP stratified by geographical region and country. The incidence rates (95% CI) of PSSP in Europe [eight studies, 1,663 participants (48%)] Oceania [two studies, 1,700 participants (49%)] and America [one study, 133 participants (4%)] were 0.31 (95% CI 0.18–0.44), 0.23 (95% CI 0.12–0.34), and 0.24 (95% CI 0.17–0.31), respectively. Meanwhile, the prevalence rates (95% CI) of PSSP in Europe [five studies, 3,052 participants (82%)], Asia [three studies, 501 participants (14%)], America [one study, 46 participants (1%)] and Africa [one study, 102 participants (3%)] were 0.14 (95% CI 0.06–0.21), 0.50 (95% CI 0.25–0.76), 0.37 (95% CI 0.23–0.51), and 0.75 (95% CI 0.67–0.84), respectively. The incidence rates (95% CI) of PSSP in the UK, which had the highest number of included studies [six studies, 1,305 participants (37%)], was 0.32 (95% CI 0.15–0.49). The incidence in New Zealand, which had the highest number of participants among the included studies [one study, 1,474 participants (42%)], was 0.17 (95% CI 0.15–0.19). The prevalence rates (95% CI) of PSSP in Sweden [one study, 140 participants (4%)] and Nigeria [one study, 102 participants (3%)] were 0.06 (95% CI 0.02–0.10) and 0.75 (95% CI 0.67–0.84), respectively, which were the lowest and highest prevalence rates in the included studies. The prevalence in Poland, which had the highest number of participants among the cross-sectional studies [one study, 1,075 participants (29%)], was 0.15 (95% CI 0.12–0.17).

**Table 5:** Subgroups analysis of incidence and prevalence PSSP.

**Subgroups**	**Number of studies**	**Number of participants**	**Meta-analysis**	**95%CI**	***P*-value**	***I* ^**2**^**
**Incidence of PSSP**						
Main analysis	11	3,496	0.29	(0.21, 0.36)	*p* = 0.000	97.3%
**Geographical region**						
Europe	8	1,663	0.31	(0.18, 0.44)	*p* = 0.000	97.7%
Oceania	2	1,700	0.23	(0.12, 0.34)	*p* = 0.000	92.3%
America	1	133	0.24	(0.17, 0.31)	*	*
**Country**						
UK	6	1,305	0.32	(0.15, 0.49)	*p* = 0.000	98.2%
Sweden	1	327	0.22	(0.17, 0.26)	*	*
Netherlands	1	31	0.35	(0.19, 0.52)	*	*
New Zealand	1	1,474	0.17	(0.15, 0.19)	*	*
Australia	1	226	0.29	(0.23, 0.35)	*	*
Canada	1	133	0.24	(0.17, 0.31)	*	*
**Prevalence of PSSP**						
Main analysis	10	3,701	0.33	(0.22, 0.43)	*p* = 0.000	98.8%
**Geographical region**						
Europe	5	3,052	0.14	(0.06, 0.21)	*p* = 0.000	97.7%
Asia	3	501	0.50	(0.25, 0.76)	*p* = 0.000	97.1%
America	1	46	0.37	(0.23, 0.51)	*	*
Africa	1	102	0.75	(0.67, 0.84)	*	*
**Country**						
Sweden	1	140	0.06	(0.02, 0.10)	*	*
Denmark	1	608	0.15	(0.12, 0.18)	*	*
Italy	1	443	0.28	(0.24, 0.33)	*	*
Poland	1	1,075	0.15	(0.12, 0.17)	*	*
France	1	786	0.04	(0.03, 0.05)	*	*
Turkey	2	272	0.62	(0.56, 0.68)	*P* = 0.685	0.0%
Korea	1	229	0.27	(0.21, 0.33)	*	*
USA	1	46	0.37	(0.23, 0.51)	*	*
Nigeria	1	102	0.75	(0.67, 0.84)	*	*

*not appropriate.

**Figure 3:**
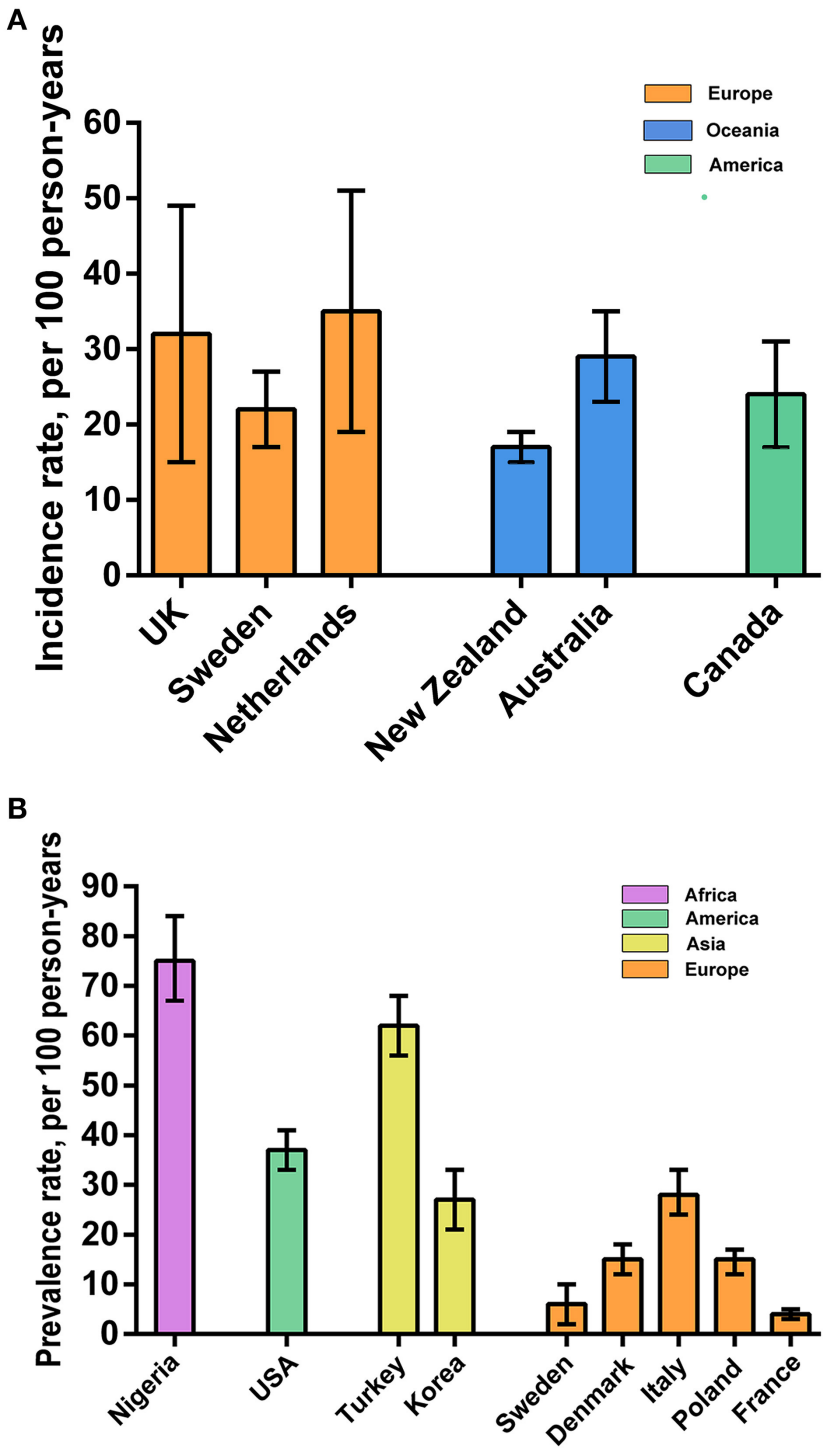
Geographical regions. **(A)** Incidence of PSSP on different geographical regions; **(B)** Prevalence of PSSP on different geographical regions.

Figures 3A,B show the distribution of the reported incidence and prevalence rates of PSSP across regions. The incidence and prevalence of PSSP in the different geographical regions and different countries were an average of 0.29 and 0.33, respectively.

### Annual Incidence and Prevalence Rates

**Figure 4:**
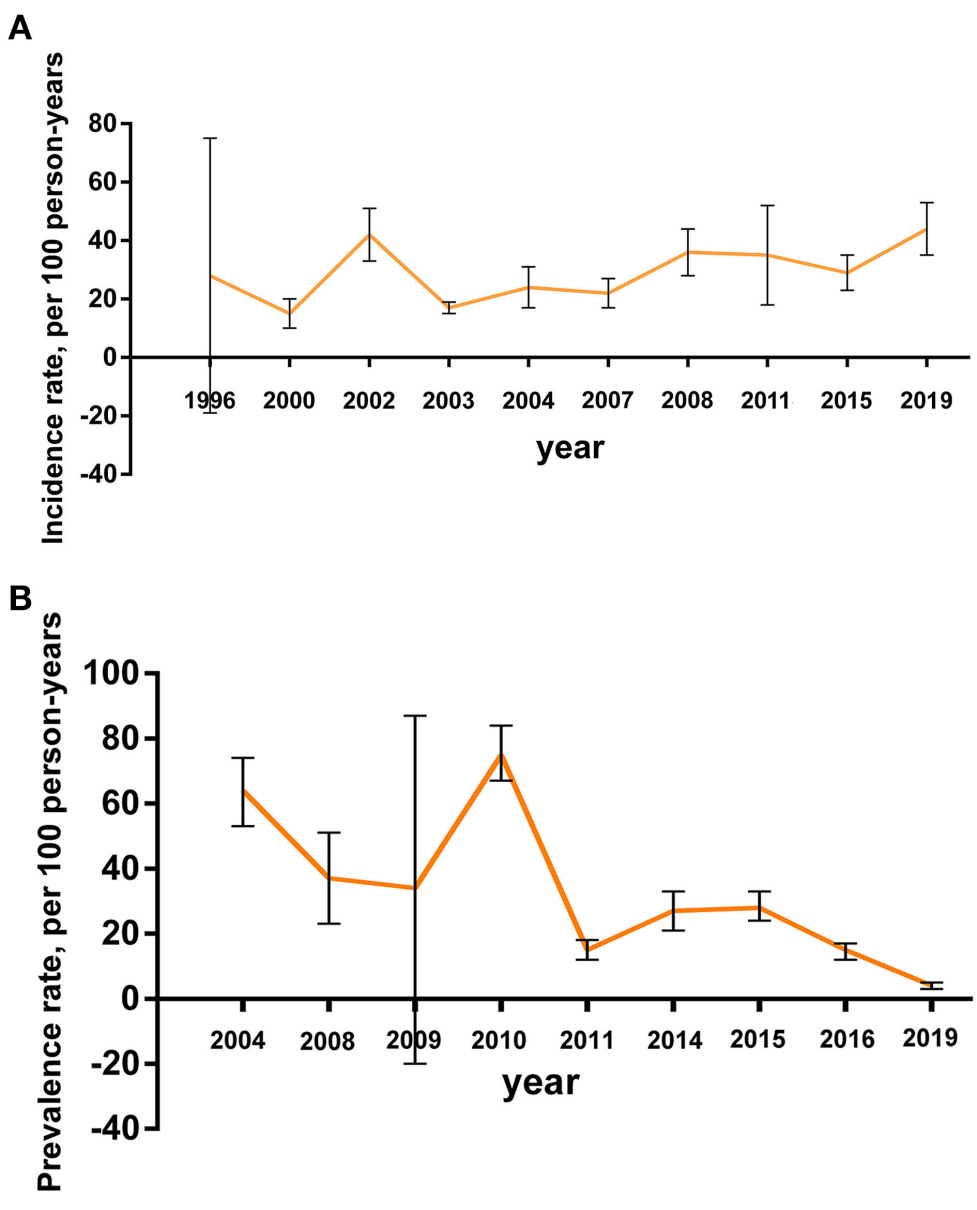
Annual trend. **(A)** The annual incidence rate of PSSP; **(B)** The annual prevalence rate of PSSP.

Broken line graphs are used to show the annual incidence and prevalence rates (and 95% CIs) of PSSP in Figures 4A,B. The annual incidence and prevalence rates in different regions, we found that the incidence of PSSP fluctuates around 0.30, and the prevalence was 0.04–0.75. The graphs also clearly showed the trend of the incidence and prevalence rates over time.

## Discussion

We reviewed the existing epidemiological evidence regarding the incidence and prevalence rates of PSSP. Our findings suggested that stroke patients have a high overall incidence (0.29) and prevalence (0.33) of PSSP. The incidence and prevalence of PSSP in the included studies varied significantly. For the incidence of PSSP, Davenport et al. (18) showed that the incidence of PSSP was 0.04, but Wanklyn et al. (19) found an incidence of 0.53. Lindgren et al. (24), Ratnasabapathy et al. (26) and Adey-Wakeling et al. (27) conducted population-based studies and found that the incidence of PSSP ranged from 0.15 to 0.29. Menoux et al. (37) and Lundström et al. (33) found prevalence rates of 0.04 and 0.06, respectively, while Hamzat et al. (28) found a prevalence rate of 0.75. Klit et al. (34) found a prevalence rate of 0.15 based on population-based research.

The incidence rate of PSSP in Europe, Oceania, and America were consistent with the overall incidence rate (1, 2). However, the prevalence of PSSP fluctuated greatly across different geographical regions. This may be related to the level of development in regions and countries, which were similar to the development of science and technology and the technical level of disease diagnosis (39). Meanwhile, patients have a wider range of information sources, higher medical service quality and health care, and a fuller understanding of the disease, so they can receive timely treatment, which leads to lower incidence and prevalence rates (40). In addition, these findings may be related to the different races in Asia, Europe, America, Oceania and Africa. The incidence and prevalence rates also differ across climates.

The included articles were from different settings, and have included different populations. Except for four population-based studies (24, 26, 27, 34), other studies were under rehabilitation settings. As we know, PSSP is more common among persons with hemiparesis under rehabilitation than in the population, which may have impact on the results. Among the studies under rehabilitation settings, nine (17, 18, 20, 21, 29, 30, 32, 35, 37) have included stroke patients from hospitals, rehabilitation units or outpatient clinics, while other eight studies (19, 22, 23, 25, 28, 31, 33, 36) all have limited the age, function or medical history of the stroke patients. For example, Hamzat et al. (28) only recruited self-ambulant persons that could comprehend instructions, and Wanklyn et al. (19) only included patients aged over 60 years, about to be discharge home and with a Barthel score <20. The different participant selection criteria may result in higher or lower incidence/prevalence rates.

Moreover, the different PSSP inclusion criteria, assessments and evaluation time points in the included articles may also affect the incidence and prevalence. McLean (17) stated that shoulder pain was considered to be present when the patient localized discomfort to any aspect of the affected shoulder, either at rest or with passive or active movement. Davenport et al., Langhorne et al. and Janus-Laszuk et al. (18, 20, 36) defined shoulder pain as pain in the shoulder area requiring analgesia on two or more consecutive days. Nadler et al. (23) indicated that pain was a numerical score of >0 on at least one numerical rating scale (at rest, on movement, at night) and subjective report of pain on a minimum of one activity of Daily Living. Roosink et al. (25) defined PSSP as shoulder pain confined to the shoulder and/or C5 dermatome of the contralesional side with an onset after stroke and present during rest or during active or passive motion. Adey-Wakeling et al. (27) considered hemiplegic shoulder pain as any subjective complaint of pain in the contralesional, or affected hemiplegic shoulder following stroke. Menoux et al. (37) diagnosed shoulder pain through required treatment, pharmacologic or physiotherapy. Kwon et al. (32) included patients that experienced musculoskeletal pain in at least one ipsilateral upper limb joint, while Paolucci et al. (35) distinguished musculoskeletal pains (including low back pain and joint pains), shoulder pain, and spasticity-related pain in the study. Besides, Klit et al. (34) described the prevalence and pain types of new onset chronic pain, which was defined as constant or remitting pain lasting more than 3 months and with onset at or after the stroke and within the last 2 years. For the different assessments in the included studies, it can influence the diagnosis of shoulder pain, and thus the incidence of shoulder pain. For example, the result of self-report and questionnaire by the patients was related to the patients' response to pain. By contrast, clinical assessments, VAS, NRS and BNS were evaluated by specialists, which were better objective and accurate. But the result of assessment has a relationship with the patients' response and specialists skills as well. Sensitive or in sensitive to pain and the clinical skills good or not may make the incidence and/or prevalence rate of PSSP higher or lower. As shown in the tables (Tables 3, 4), the time points for assessing PSSP also varied in the included studies. It may be within 72 h (23), 2 years (34) later or other time after stroke. PSSP usually occurs after stroke onset within 2 weeks−2 months (1, 2), and as the treatment progresses and time passes, PSSP may gradually disappear. Therefore, the assessment too early or too late may lead to lower incidence and prevalence.

By summarizing the annual incidence and prevalence rates in different regions, we found that the incidence of PSSP fluctuates around 0.30, and the prevalence has a downward trend over time. This indicated that PSSP was a common poststroke complication and had a high incidence rate (1, 2). However, with the continuous improvement of the overall medical level, and people's desire for medical treatment is increasing in recent years. Also, due to a variety of active intervention methods, such as NMES (38, 41–43), active and passive motor training (44–46), and intramuscular stickers (47, 48), the prevalence rate has declined. It may be related to a better understanding of the disease, increased patients' compliance, early intervention at admission, regular check-ups, and better home rehabilitation. With ongoing advances in existing technologies and the application of new technologies and drugs, patients have a better chance to recover more quickly, reduce recurrence and prevent the development of PSSP.

In addition, based on the available evidence, we also found that the incidence and prevalence of PSSP were similar in men and women, while the incidence and prevalence of right-sided hemiplegia were higher in men. Contrary to the results reported by Anwer et al. (49), some included studies (23, 25–27, 31) showed that patients with right-sided hemiplegia were more likely to have PSSP, possibly because of different included populations. There seemed to be three main etiological groups that may cause PSSP, namely central post-stroke pain, complex regional pain syndrome, and musculoskeletal pain (spasticity-related pain, shoulder subluxation induced pain, etc.) (50, 51). Similarly, many different risk factors for PSSP have also been found (49, 52). Further understanding of the etiology and risk factors of PSSP is important to minimize the risk of developing shoulder pain following a stroke.

### Limitations

Several limitations of this study should be acknowledged. First, the clinical heterogeneity of the included studies was high and publication bias was existence due to the influence of many factors, such as region, population, baseline situation and diagnostic criteria. Additionally, due to the limitations of the number of studies and unavoidable measurement bias, in-depth analysis cannot be carried out, which affected the accuracy of the results. Second, the study area was widely distributed, the age range was large, the study could not be divided into different groups, and its epidemiological characteristics could not be accurately revealed. Third, PSSP had a multifactorial etiology and a variety of treatment methods, but this study failed to analyze the impact of different etiologies and treatment methods on the incidence and prevalence, which limits the clinical significance of the study.

### Conclusions

In summary, current evidence suggests that the incidence and prevalence of PSSP are high and may be influenced by geographical region and time. Currently, there are few high-quality epidemiological studies of PSSP, as most studies on PSSP have explored its pathogenesis and the development of interventions. In the future, more systematic and comprehensive epidemiological investigations are needed to further explore different intervention times and means as well as preventive treatments for patients with PSSP to provide more accurate guidance for clinical practice.

## Data Availability Statement

The original contributions presented in the study are included in the article/Supplementary Material, further inquiries can be directed to the corresponding authors.

## Author Contributions

JL and PW assisted in study conception and design and critical revision of the article. QZ, DC, and YS helped with the search and quality appraisal. QZ and MB performed the statistical analysis and interpretation. QZ and DC constructed the figures and tables and helped draft the article. All authors contributed to the article and approved the submitted version.

## Conflict of Interest

The authors declare that the research was conducted in the absence of any commercial or financial relationships that could be construed as a potential conflict of interest.

## Publisher's Note

All claims expressed in this article are solely those of the authors and do not necessarily represent those of their affiliated organizations, or those of the publisher, the editors and the reviewers. Any product that may be evaluated in this article, or claim that may be made by its manufacturer, is not guaranteed or endorsed by the publisher.
