# Supplementary material for: Incidence and Prevalence of Poststroke Shoulder Pain Among Different Regions of the World: A Systematic Review and Meta-Analysis
Source: Front Neurol. 2021 Nov 4;12:724281. doi: 10.3389/fneur.2021.724281 (PMC8600331; doi:10.3389/fneur.2021.724281)
Supplement: Supplementary file 1 [file Presentation_1.zip › Suppl. Table 3. Complete Search Terms for PubMed.docx]

**Supplementary Table 3.** Complete Search Terms for PubMed

**A search example for Pubmed**

**Search terms：**

(((((((((((((((((((((((((((((Strokes) OR (Cerebrovascular Accident)) OR (Cerebrovascular Accidents)) OR (CVA (Cerebrovascular Accident))) OR (CVAs (Cerebrovascular Accident))) OR (Cerebrovascular Apoplexy)) OR (Apoplexy, Cerebrovascular)) OR (Vascular Accident, Brain)) OR (Brain Vascular Accident)) OR (Brain Vascular Accidents)) OR (Vascular Accidents, Brain)) OR (Cerebrovascular Stroke)) OR (Cerebrovascular Strokes)) OR (Stroke, Cerebrovascular)) OR (Strokes, Cerebrovascular)) OR (Apoplexy)) OR (Cerebral Stroke)) OR (Cerebral Strokes)) OR (Stroke, Cerebral)) OR (Strokes, Cerebral)) OR (Stroke, Acute)) OR (Acute Stroke)) OR (Acute Strokes)) OR (Strokes, Acute)) OR (Cerebrovascular Accident, Acute)) OR (Acute Cerebrovascular Accident)) OR (Acute Cerebrovascular Accidents)) OR (Cerebrovascular Accidents, Acute)) OR ((((((((((((((((((((((((((((Hemiplegias) OR (Hemiplegia, Transient)) OR (Hemiplegias, Transient)) OR (Transient Hemiplegia)) OR (Transient Hemiplegias)) OR (Monoplegia)) OR (Monoplegias)) OR (Hemiplegia, Post-Ictal)) OR (Hemiplegia, Post Ictal)) OR (Hemiplegias, Post-Ictal)) OR (Post-Ictal Hemiplegia)) OR (Post-Ictal Hemiplegias)) OR (Hemiplegia, Crossed)) OR (Crossed Hemiplegia)) OR (Crossed Hemiplegias)) OR (Hemiplegias, Crossed)) OR (Hemiplegia, Flaccid)) OR (Flaccid Hemiplegia)) OR (Flaccid Hemiplegias)) OR (Hemiplegias, Flaccid)) OR (Hemiplegia, Infantile)) OR (Hemiplegias, Infantile)) OR (Infantile Hemiplegia)) OR (Infantile Hemiplegias)) OR (Hemiplegia, Spastic)) OR (Hemiplegias, Spastic)) OR (Spastic Hemiplegia)) OR (Spastic Hemiplegias))) AND (((Pain, Shoulder) OR (Pains, Shoulder)) OR (Shoulder Pains))
