# Supplementary figures and images for: Incidence and Prevalence of Poststroke Shoulder Pain Among Different Regions of the World: A Systematic Review and Meta-Analysis
Source: Front Neurol. 2021 Nov 4;12:724281. doi: 10.3389/fneur.2021.724281 (PMC8600331; doi:10.3389/fneur.2021.724281)

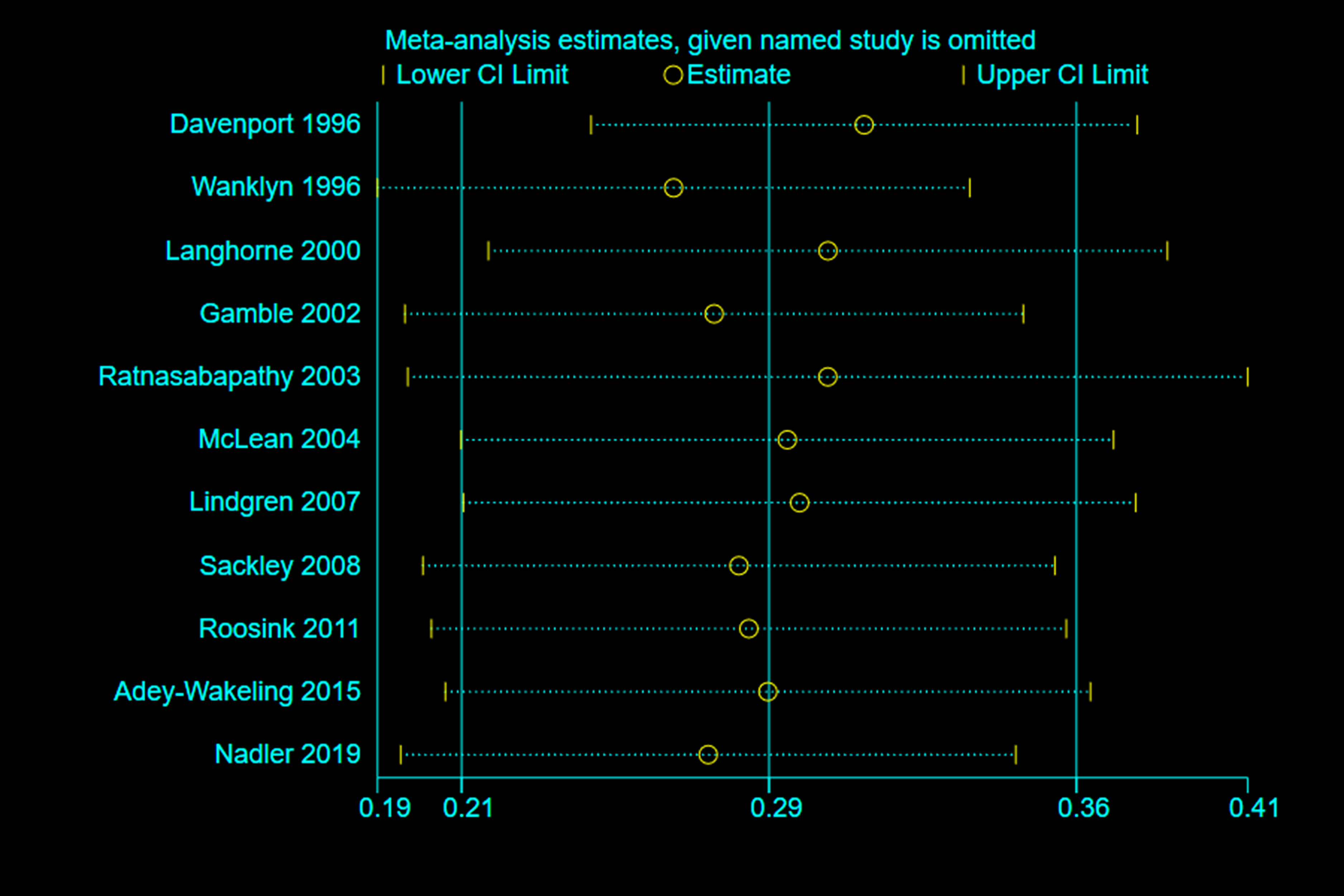

Supplement: Supplementary file 2 [file Presentation_2.zip › Suppl. Fig.1(A) Sensitivity analysis of incidence PSSP.tif]

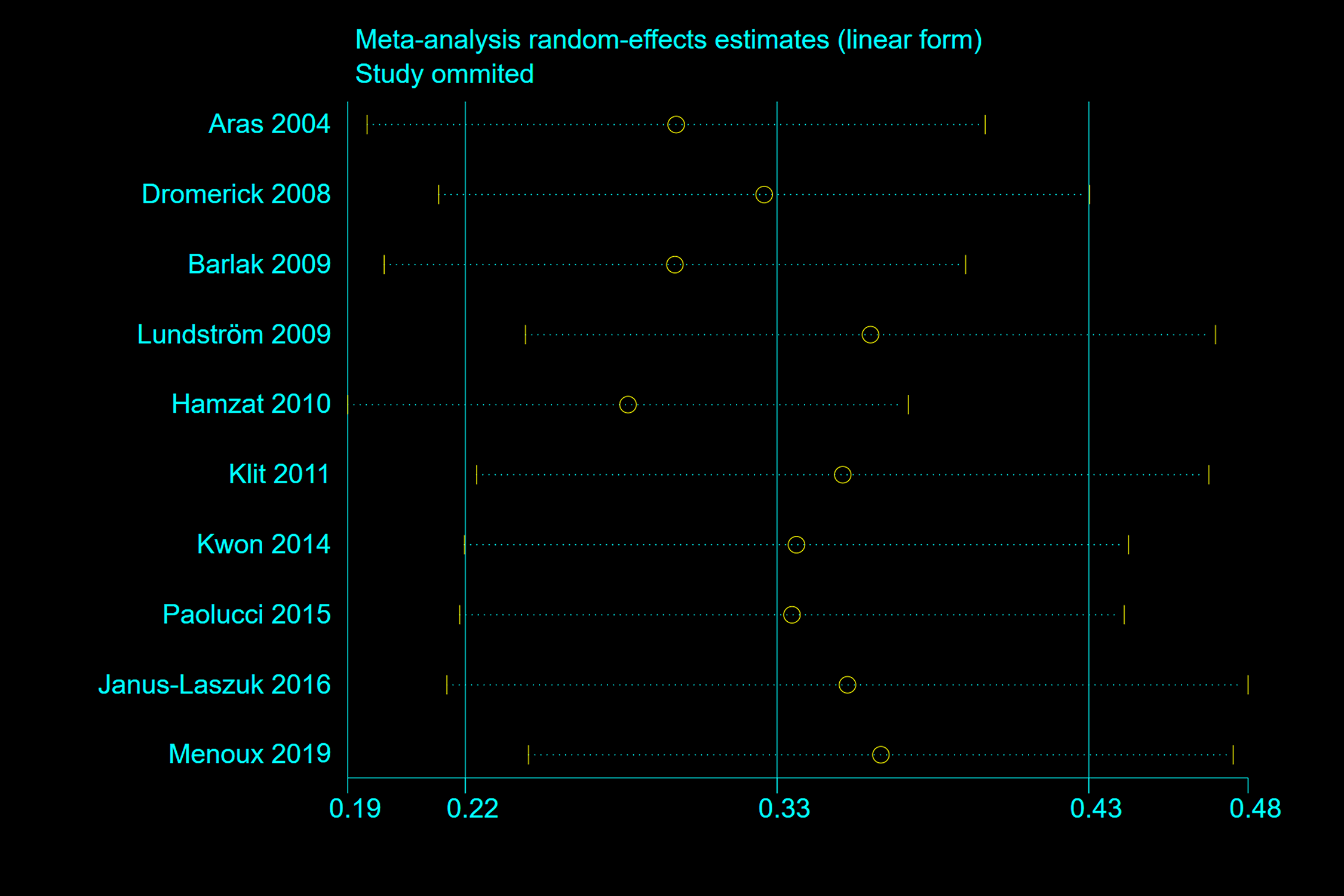

Supplement: Supplementary file 2 [file Presentation_2.zip › Suppl. Fig.1(B) Sensitivity analysis of prevalence PSSP.tif]

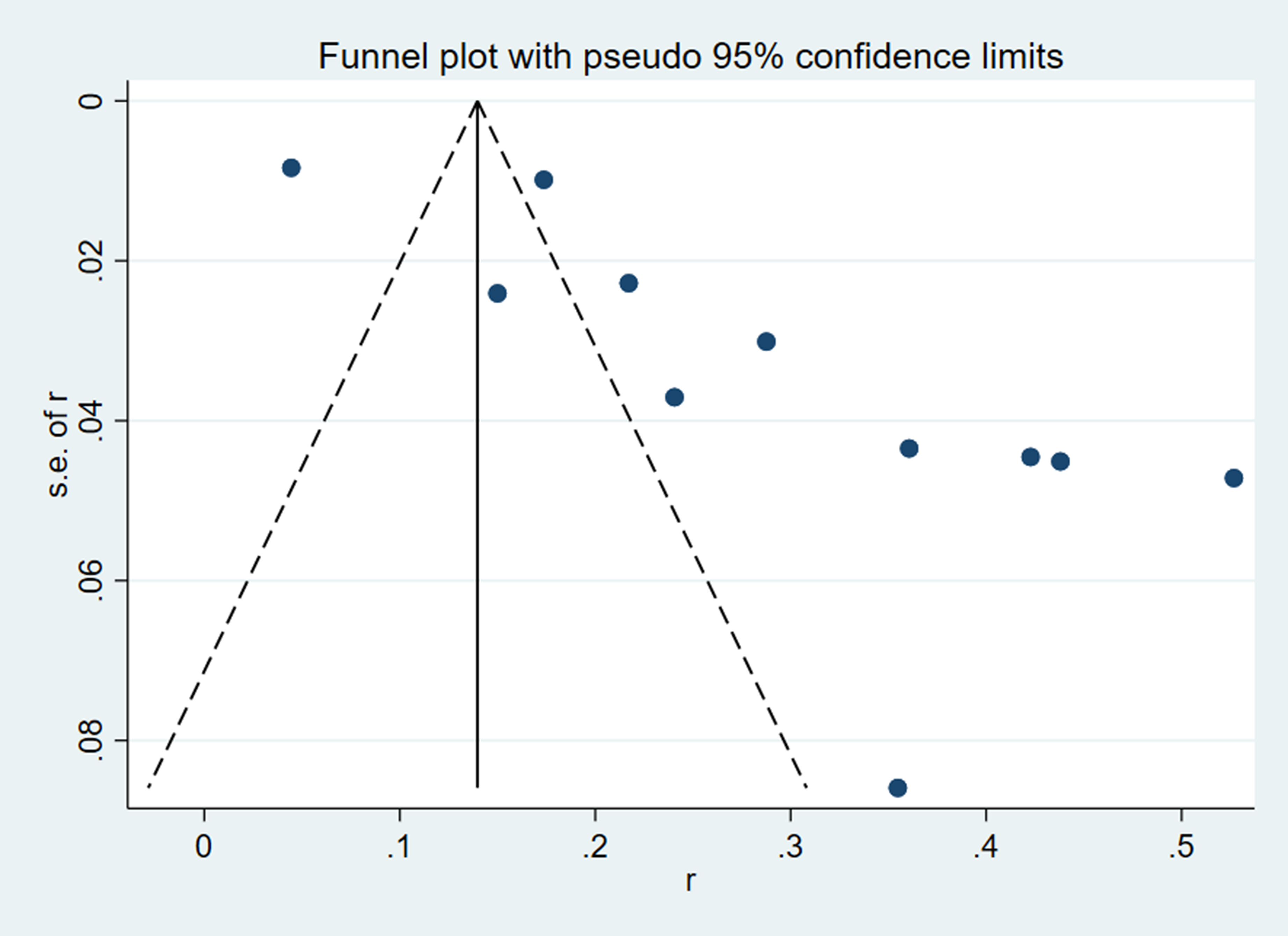

Supplement: Supplementary file 2 [file Presentation_2.zip › Suppl. Fig.2(A) Funnel plots of incidence of PSSP.tif]

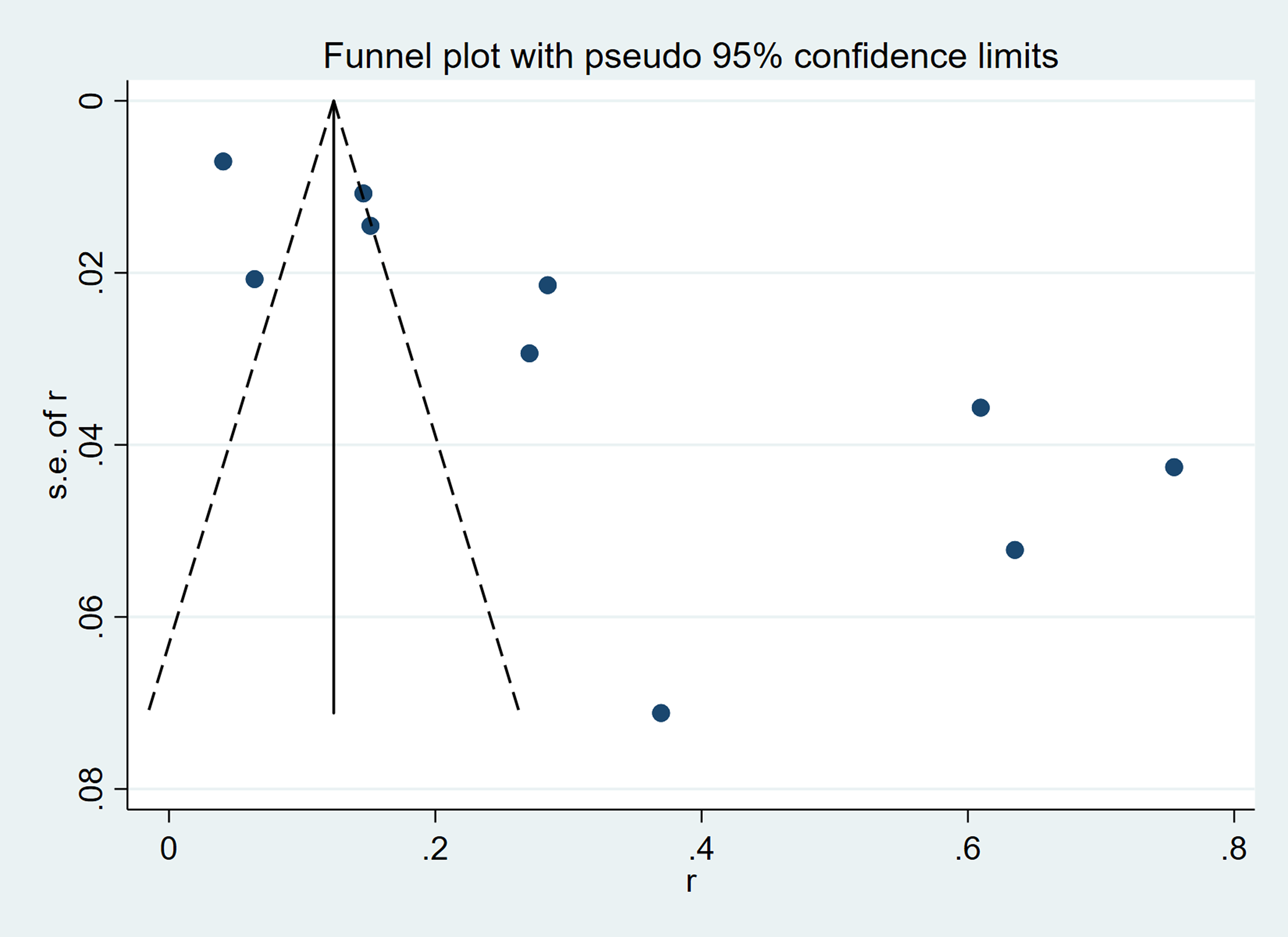

Supplement: Supplementary file 2 [file Presentation_2.zip › Suppl. Fig.2(B) Funnel plots of prevalence of PSSP.tif]
